# Association of prehospital pupillary diameter with return of spontaneous circulation and neurological outcome after out-of-hospital cardiac arrest: A multicenter retrospective analysis

**DOI:** 10.1016/j.resplu.2025.101112

**Published:** 2025-09-26

**Authors:** Munekazu Takeda, Ryokan Ikebe, Takuya Oshiro, Mizuho Namiki, Shimpei Asada, Shusuke Mori, SOS-KANTO 2017 Study Group

**Affiliations:** Department of Emergency and Critical Care Medicine, Tokyo Women’s Medical University, 8-1, Kawada-cho, Shinjuku-ku, Tokyo 162-8666, Japan

**Keywords:** Pupillary diameter, Out-of-hospital cardiac arrest, Emergency medical services, Return of spontaneous circulation, Prognostication

## Abstract

**Background:**

In Japan, emergency medical services (EMS) routinely record pupillary size and pupillary light reflex (PLR) during prehospital care for out-of-hospital cardiac arrest (OHCA). While hospital-based studies have established the prognostic value of pupillary findings, the significance of prehospital pupillary diameter remains uncertain.

**Objective:**

To examine whether pupillary diameter at EMS contact predicts prehospital return of spontaneous circulation (ROSC) and 30-day neurological outcomes.

**Methods:**

This retrospective cohort study analyzed SOS-KANTO 2017, a prospective multicenter OHCA registry. Of 9909 adults, 8494 were eligible after excluding those not in arrest at EMS contact or with missing data. EMS personnel, trained in neurological assessment, documented pupillary diameter using standardized visual charts (0.5-mm increments) but recorded registry values in 1-mm categories (1–8 mm). The primary outcome was prehospital return of spontaneous circulation (ROSC), and the secondary outcome was 30-day favorable neurological status (CPC 1–2). Multivariable logistic regression adjusted for demographics, resuscitation factors, and Utstein variables. Receiver operating characteristic (ROC) analyses, treating failure to achieve ROSC as the positive condition, were performed to assess sensitivity, specificity, and false positive rate (FPR) for futility thresholds.

**Results:**

Larger pupillary diameter was independently associated with reduced odds of favorable 30-day outcome (odds ratio [OR] per 1-mm increase, 0.73; 95 % CI 0.61–0.86; *p* < 0.001). Pupillary diameter was also inversely associated with achieving ROSC (OR per 1-mm increase, 0.694; 95 % CI 0.644–0.748; *p* < 0.001). Thresholds of ≥7–8 mm predicted failure to achieve ROSC with high specificity (0.93–0.99) but poor sensitivity.

**Conclusions:**

Prehospital pupillary diameter is independently associated with both ROSC and 30-day neurological outcome. Although extreme dilation (≥7–8 mm) provides a highly specific marker of futility, low sensitivity precludes its use as a stand-alone criterion. Pupillary assessment may nonetheless contribute, in combination with other prehospital indicators, to a multimodal framework for early decision-making.

## Introduction

Cardiac arrest remains a major cause of death and disability worldwide, with survival rates and neurological outcomes varying significantly despite advances in resuscitation.^1^, ^2^ Rapid and accurate early prognostication is crucial for directing care, communicating with families, and guiding resource allocation.^2^, ^3^

In Japan, EMS teams routinely assess multiple vital signs, including pupillary size and pupillary light reflex (PLR), during prehospital care. The Japanese Resuscitation Council (JRC) 2020 guidelines recommend using quantitative pupillary measurements 72 h after return of spontaneous circulation (ROSC) to predict neurological outcome in comatose patients.^4^ However, the prognostic value of pupillary diameter documented during the prehospital phase remains uncertain.^5^, ^6^

Pupillary size and reactivity reflect brainstem function and cerebral perfusion and are regarded as critical neurological signs after cardiac arrest.^6^, ^7^, ^8^ Manual assessment of PLR with a penlight is subject to observer variability and environmental interference, leading to potential inaccuracies.^7^, ^9^ Automated pupillometry, by contrast, provides objective quantification and has been shown to improve outcome prediction in both in-hospital and out-of-hospital cardiac arrest cohorts.^5^, ^9^, ^10^ For example, Yokobori et al. reported that quantitative pupillometry and neuron-specific enolase independently predicted ROSC following cardiogenic OHCA,^11^ while Tamura et al. demonstrated in a multicenter study that early pupillary parameters predicted neurological outcomes after OHCA.^12^ Similarly, Riker et al. found that the neurological pupil index and quantitative PLR predicted outcome early after arrest,^13^ and Nyholm et al. recently validated pupillometry thresholds for neuroprognostication in a large randomized trial substudy.^14^ Even during active resuscitation, pupillometry may be feasible, as shown in case reports using infrared devices.^15^

Although several hospital-based studies have explored the utility of pupillary findings for prognosis, evidence for their value when assessed by EMS at the scene of arrest is lacking.^6^, ^8^, ^16^ Understanding whether prehospital pupillary diameter can predict outcomes would provide a valuable tool for early triage and decision-making.^3^

In this study, we investigate the relationship between prehospital pupil size and reactivity with return of spontaneous circulation, and subsequent neurological outcomes.

## Methods

### Study design and population

This study was a retrospective cohort analysis of data from the SOS-KANTO 2017 registry, a multicenter, prospective, observational database of patients with out-of-hospital cardiac arrest (OHCA) in Japan. The registry prospectively enrolled consecutive OHCA patients transported by emergency medical services (EMS) between January 2017 and December 2018 across 67 participating hospitals and their affiliated EMS agencies in the Kanto region.

Standardized case report forms were completed in real time by EMS personnel at the scene or during transport, using the nationally standardized ambulance record system. This system specifies required data elements, including patient demographics, prehospital interventions, clinical variables, and neurological assessments. EMS providers recorded pupillary diameter and light reflex as part of the standard neurological examination protocol, for which they receive formal training during prehospital care certification. All forms were reviewed for completeness before submission to the registry.

**Fig. 1:**
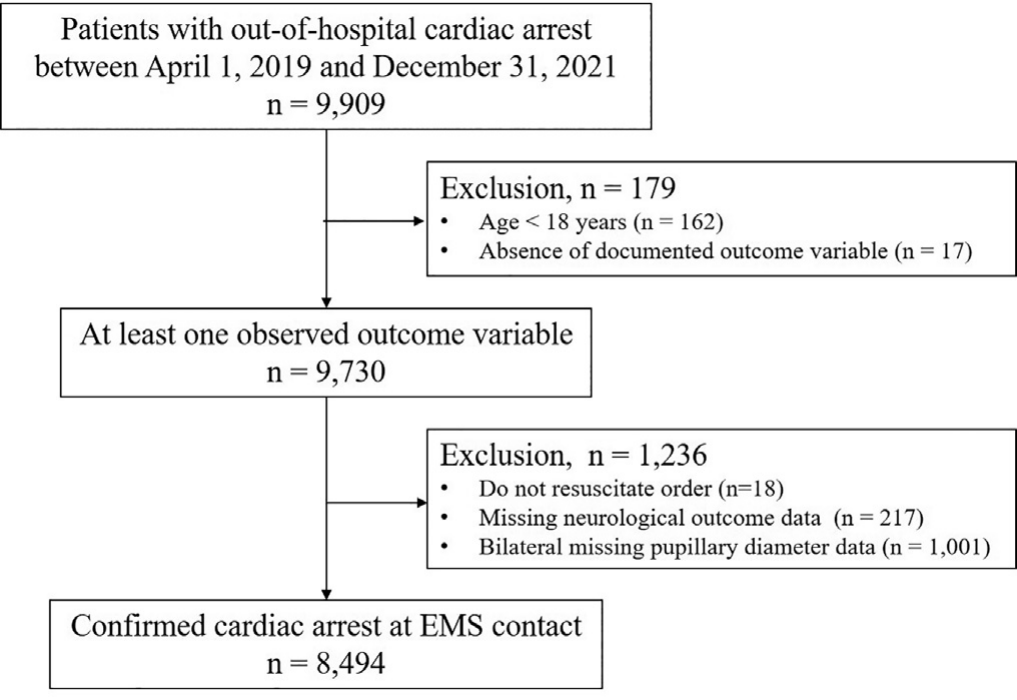
**Study flow diagram of patient selection from the SOS-KANTO 2017 registry.** A total of 9909 patients with out-of-hospital cardiac arrest between January 2017 and December 2018 were initially screened. After excluding 179 patients (age <18 years, *n* = 162; absence of documented outcome variable, *n* = 17), 9730 patients with at least one observed outcome variable remained. A further 1236 patients were excluded due to do-not-resuscitate (DNR) orders (*n* = 18), missing neurological outcome data (*n* = 217), or bilateral missing pupillary diameter data (*n* = 1001). The final analytic cohort comprised 8494 patients with confirmed cardiac arrest at EMS contact.

Of the 9909 patients registered in the SOS-KANTO 2017 database, 179 were excluded because they were younger than 18 years (*n* = 162) or had no documented outcome variable (*n* = 17). This left 9730 patients with at least one observed outcome variable. A further 1236 patients were excluded due to do-not-resuscitate (DNR) orders (*n* = 18), missing neurological outcome data (*n* = 217), or bilateral missing pupillary diameter data (*n* = 1001). For patients with unilateral missing pupillary data, the available eye was used. The final analytic cohort, therefore, comprised 8494 patients with confirmed cardiac arrest at EMS contact (Fig. 1).

### Data collection and variables

EMS recorded demographic data (age, sex), prehospital factors (bystander CPR, AED shocks, initial ECG rhythm), clinical variables (body temperature, airway management, adrenaline use, venous access, site of occurrence). Neurological findings—including pupillary diameter and pupillary light reflex (PLR)—were documented at first EMS contact as required elements on the standardized ambulance record form.

### Pupillary diameter assessment

In Japan, pupillary diameter is routinely recorded by emergency medical service (EMS) personnel at the first patient contact using a nationally standardized ambulance record form. The SOS-KANTO 2017 registry specifies pupil size documentation in 1-mm categories ranging from 1 to 8 mm to ensure uniformity across all participating sites and to minimize misclassification bias.

**Fig. 2:**
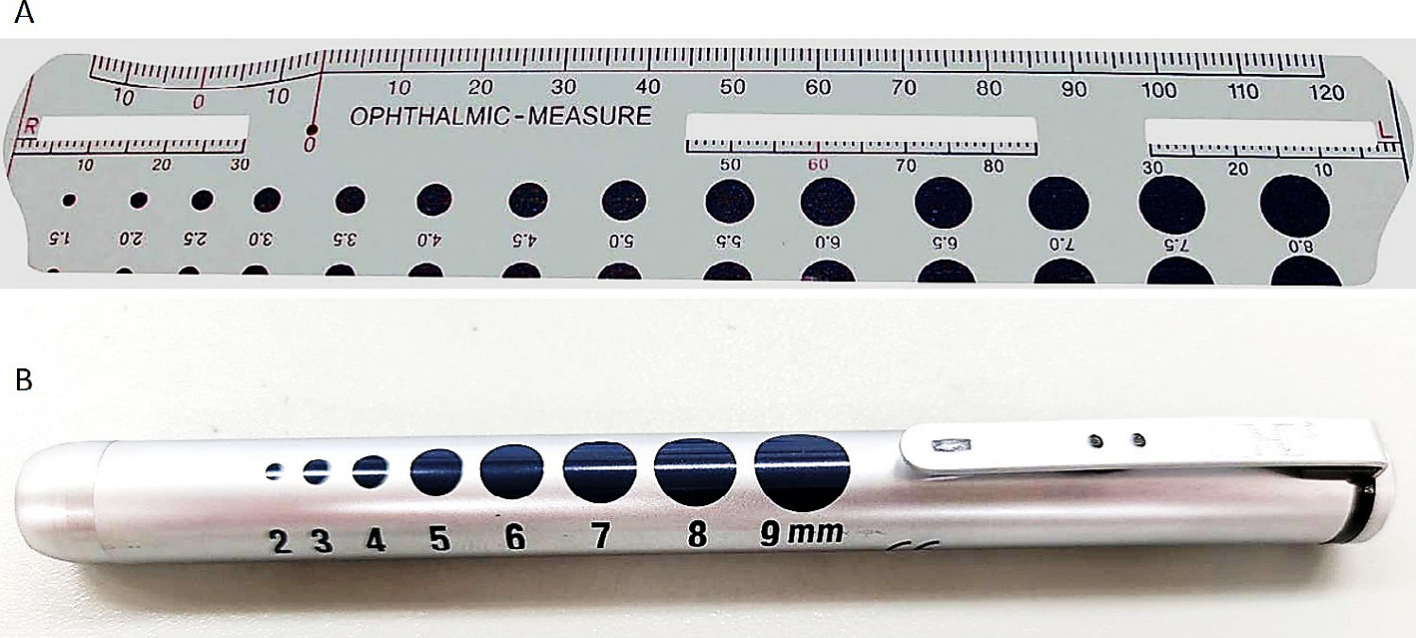
**Tools used by EMS providers for pupillary diameter assessment.**(A) A printed pupillary gauge chart (1.5–8.0 mm in 0.5-mm increments) used in some EMS regions to aid visual estimation. (B) A penlight-based gauge with markings from 2–9 mm in 1-mm increments.For the SOS-KANTO 2017 registry, however, the official recorded value was always rounded to the nearest 1 mm and documented within the standardized 1–8 mm scale.

**Fig. 3:**
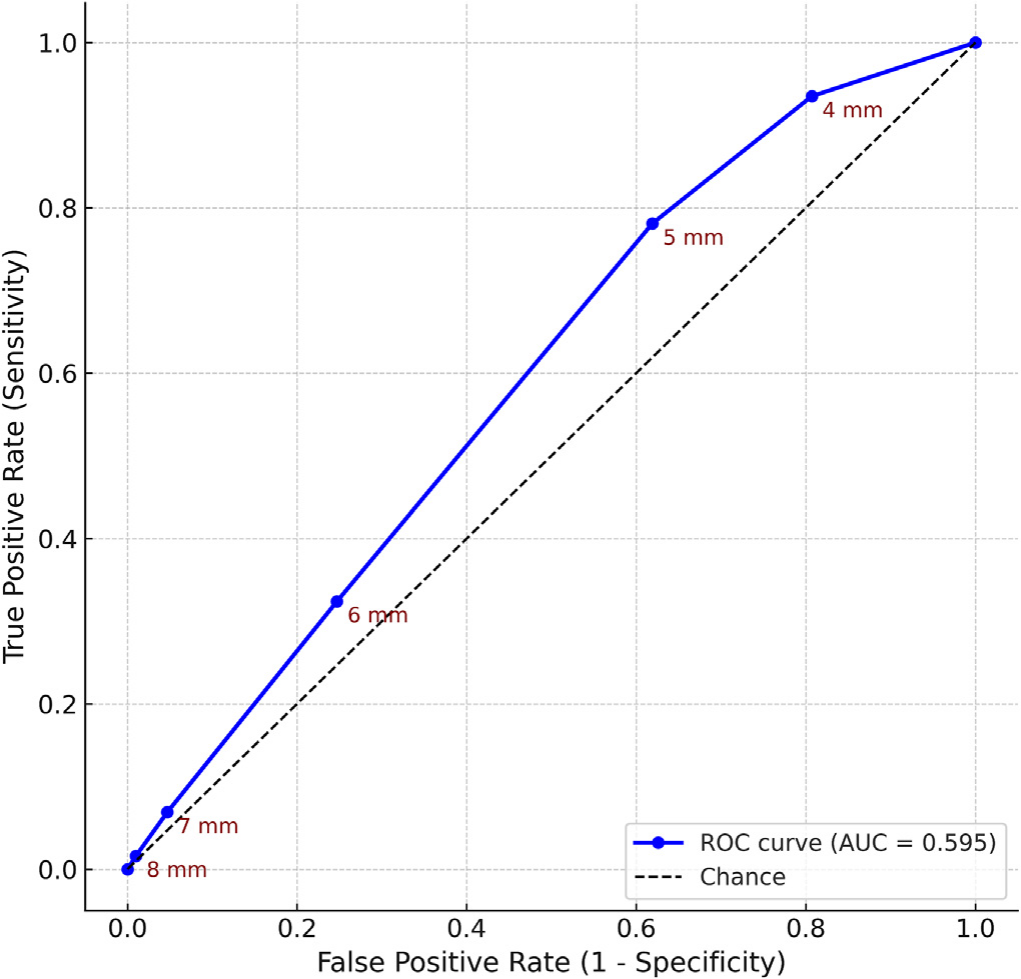
**Receiver operating characteristic (ROC) curve for pupillary diameter cutoffs in predicting failure to achieve prehospital return of spontaneous circulation (ROSC).** The ROC curve was generated using pupillary diameter thresholds documented at EMS contact (4–8 mm). Sensitivity (true positive rate) is plotted against 1–specificity (false positive rate). Each cutoff value is annotated on the curve. The overall discriminatory ability of pupillary diameter was modest (AUC = 0.595). Larger thresholds improved specificity at the expense of sensitivity: cutoffs of ≥7 mm and ≥8 mm achieved specificities of 0.953 and 0.990, but sensitivities declined to 0.069 and 0.016, respectively.

In some EMS regions, visual aids such as a printed pupillary gauge (Fig. 2A, 1.5–8.0 mm in 0.5-mm increments) or a penlight-based gauge (Fig. 2, Fig. 2, Fig. 3B, 2–9 mm in 1-mm increments) were available to assist manual estimation. However, for registry purposes, the official recorded value was always rounded to the nearest 1 mm and documented within the predefined 1–8 mm scale.

All EMS providers receive formal training in neurological assessment, including pupillary diameter estimation and evaluation of the pupillary light reflex (PLR), as part of their prehospital care certification process. When asymmetry was present between both eyes, the larger value was used for analysis.

Pupillary measurements obtained after hospital arrival or in cases where documentation explicitly indicated that assessment occurred following ROSC were excluded from the primary analysis. EMS providers were instructed to record pupil size at first patient contact, typically during a brief pause in chest compressions. However, because the exact timing relative to ongoing CPR was not uniformly documented, some residual uncertainty regarding measurement timing may remain.

### Pupillary light reflex (PLR)

PLR was assessed by direct illumination with a penlight and recorded as “present” or “absent” on the standardized form. Pupillary size and PLR were documented for at least one eye using the standardized ambulance record form. In cases with unilateral documentation, the available side was used for analysis. In our cohort, 40 patients (0.4 %) had only right-eye documentation and 25 patients (0.3 %) had only left-eye documentation, while 8557 patients (86.4 %) had bilateral data.

### Timing of assessment

Pupillary assessment was generally performed during a brief pause in chest compressions or immediately after ROSC, in accordance with local EMS protocols. The exact timing relative to ongoing CPR was not uniformly documented and is addressed as a study limitation.

### Variable selection for multivariate analysis

A total of 39 variables were evaluated descriptively. Of these, 32 variables were eligible for univariate logistic regression. Variables with > 10 % missingness were excluded from multivariate modeling. Multivariable models were then constructed using covariates identified as clinically relevant and supported by prior literature, including age, sex, bystander CPR, public AED use, initial rhythm, adrenaline administration, and pupillary findings.

### Outcome measures

Neurological outcome was assessed using the Cerebral Performance Category (CPC) scale (1 = good cerebral performance, 2 = moderate disability, 3 = severe disability, 4 = coma or vegetative state, 5 = brain death or death). Outcomes were dichotomized into favorable (CPC 1–2) and unfavorable (CPC 3–5). CPC scores were prospectively determined by the attending physicians at each participating hospital at 30 days after the event (or at hospital discharge if earlier) and documented on standardized hospital case report forms, which were subsequently submitted to the registry office. Patients were dichotomized into two groups: favorable outcome (CPC 1–2) and unfavorable outcome (CPC 3–5).

The primary outcome was prehospital return of spontaneous circulation (ROSC). The secondary outcome was 30-day neurological outcome, dichotomized into favorable (CPC 1–2) and unfavorable (CPC 3–5). Accordingly, we first analyzed the association between pupillary diameter and prehospital ROSC. In a secondary analysis restricted to patients who achieved ROSC, we evaluated its association with 30-day favorable neurological outcome.

The primary predictor was maximum pupillary diameter at EMS contact (per 1-mm increase), with adjustments for age, sex, bystander CPR, public AED defibrillation, rescue team defibrillation (≥1 shock), adrenaline administration, and initial rhythm (shockable VF/VT, PEA, asystole; reference = sinus/other). Pupillary diameter was also analyzed as ordered categories (1–2, 3–4, 5–6, 7, and 8 mm; reference = 3–4 mm).

For cutoff analyses, we treated failure to achieve ROSC as the positive condition and calculated sensitivity, specificity, and false positive rate (FPR) for integer cutoffs (4–8 mm), overall and stratified by pupillary light reflex (PLR) status.

### Statistical analysis

Continuous variables are reported as means (standard deviation [SD]) or medians (interquartile range [IQR]), and categorical variables as counts (%). Missing outcome data were imputed using the Last Observation Carried Forward (LOCF) method; if only 30-day survival was available, patients who died were imputed as CPC 5.

Analyses included:

1. **Univariate logistic regression** for each variable and outcome.
2. **Multivariate logistic regression**, starting with all variables with <10 % missingness, followed by stepwise reduction using the Akaike Information Criterion (AIC). Odds ratio (OR) and 95 % confidence intervals (CI) were calculated.
3. **Association with ROSC**: Logistic regression assessing the relationship between initial pupillary diameter and prehospital or in-hospital ROSC.
4. **Cut-off analysis**: Receiver operating characteristic (ROC) curve analysis to determine the optimal pupillary diameter cut-off for predicting failure to achieve ROSC, using Youden’s index. Sensitivity, specificity, and false positive rate (FPR) were calculated.
5. **Subgroup analyses**: Stratification by PLR status (present vs. absent) and extreme value analysis (<2 mm or >6 mm) to minimize potential misclassification bias.
6. **Multivariable models** including key prehospital and in-hospital interventions (bystander CPR, public AED shocks, rescue team defibrillation, adrenaline administration, hospital ROSC).

All analyses were conducted using R version 3.6.2 (R Foundation for Statistical Computing, Vienna, Austria). Statistical significance was set at *p* < 0.05.

## Results

### Cohort characteristics

Of 9909 patients in the SOS-KANTO 2017 registry, 8494 met all eligibility criteria after excluding those aged <18 years, lacking confirmed cardiac arrest at EMS contact, or missing either right-eye pupillary diameter or outcome data (Fig. 1). The mean age was 72.3 ± 16.5 years, with a median of 76 (IQR 64–84). Males comprised 61.2 % of the cohort. The average initial pupillary diameter at EMS contact was 5.07 ± 1.07 mm (median 5 mm, IQR 5–6 mm, range 1–8 mm). Among the analytic cohort, pupillary diameter was available bilaterally in 8557 patients (86.4 %), only for the right eye in 40 patients (0.4 %), and only for the left eye in 25 patients (0.3 %). In cases with unilateral documentation, the available side was used for analysis. Public AED defibrillation was rare (mean 0.025 shocks per patient), and the mean Glasgow Coma Scale score after hospital arrival was 3.08 ± 0.82 (Table 1).

**Table 1:** Baseline characteristics of patients with out-of-hospital cardiac arrest (OHCA) in the SOS-KANTO 2017 registry.

**Variable**	**N**	**Mean**	**SD**	**Median**	**Q1**	**Q3**	**Min**	**Max**
Age (years)	8494	72.3	16.5	76	64	84	18	111
Public AED shocks (n)	8494	0.025	0.23	0	0	0	0	6
Pupillary diameter right (mm)	7751	5.07	1.07	5	5	6	1	8
Pupillary diameter left (mm)	7739	5.07	1.07	5	5	6	1	8
GCS after hospital arrival	8494	3.08	0.82	3	3	3	3	15

Analyses used the larger value when both eyes were available or the documented side if unilateral. Values are presented as mean ± SD, median (IQR), or n (%), as appropriate. SD = standard deviation; AED = automated external defibrillator; GCS = Glasgow Coma Scale; IQR = interquartile range.

### Distribution of pupillary diameter

Among the 7751 patients with available right-eye measurements, the most frequently observed diameter was 5 mm (34.2 %), followed by 6 mm (23.4 %) and 4 mm (18.8 %). Smaller diameters (1–3 mm) and larger diameters (7–8 mm) were less common, together accounting for < 25 % of all cases. This distribution pattern is consistent with the use of standardized 1-mm recording bins across EMS agencies (Table 2).

**Table 2:** Distribution of pupillary diameter recorded by EMS personnel at first contact.

**Pupillary Diameter (mm)**	**N**	**% (of 7751)**
1	69	0.9 %
2	279	3.6 %
3	682	8.8 %
4	1461	18.8 %
5	2651	34.2 %
6	1811	23.4 %
7	688	8.9 %
8	110	1.4 %

Pupillary diameter was recorded at EMS contact using standardized ambulance record forms. EMS = emergency medical services.

### Association of pupillary diameter with ROSC

We next evaluated whether prehospital pupillary diameter could predict the inability to achieve ROSC. In the multivariable logistic regression analysis, larger pupils at EMS contact were independently associated with lower odds of achieving prehospital ROSC (OR per 1-mm increase, 0.694; 95 % CI 0.644–0.748; *p* < 0.001). Age was also inversely associated with ROSC (OR per year = 0.994; 95 % CI 0.989–0.999; *p* = 0.027), while sex showed no significant effect (female vs male: OR 0.857; 95 % CI 0.718–1.023; *p* = 0.087).

Several prehospital interventions were significantly related to outcomes. Bystander CPR was positively associated with ROSC (OR 1.36, 95 % CI 1.16–1.61; *p* < 0.001), as were public AED shocks (OR 2.03, 95 % CI 1.31–3.14; *p* = 0.001) and rescue team defibrillation (OR 1.51, 95 % CI 1.12–2.03; *p* = 0.007). Adrenaline administration showed a strong association with ROSC in the regression model (OR 2.68, 95 % CI 2.27–3.16; *p* < 0.001).

With respect to initial cardiac rhythm, shockable rhythms (VF/VT) were not significantly associated with ROSC (OR 1.19, 95 % CI 0.74–1.91; *p* = 0.475), whereas pulseless electrical activity showed no clear association (OR 1.23, 95 % CI 0.84–1.80; *p* = 0.280). Initial asystole, however, was strongly associated with failure to achieve ROSC (OR 0.34, 95 % CI 0.23–0.49; *p* < 0.001) (Table 3).

**Table 3:** Multivariable logistic regression analysis of factors associated with prehospital return of spontaneous circulation (ROSC achieved = 1).

**Label**	**OR**	**95 % CI low**	**95 % CI high**	**p value**
Intercept	0.835	0.43	1.62	0.593
Pupillary diameter (per 1 mm)	0.694	0.644	0.748	<0.001
Age (per year)	0.994	0.989	0.999	0.027
Female sex (vs male)	0.857	0.718	1.023	0.087
Bystander CPR (yes)	1.363	1.155	1.609	<0.001
Public AED shock (yes)	2.03	1.312	3.14	0.001
Rescue team defibrillation (yes)	1.508	1.117	2.034	0.007
Adrenaline (yes)	2.678	2.268	3.162	<0.001
Initial rhythm shockable (VF/VT)	1.189	0.74	1.91	0.475
Initial rhythm PEA	1.233	0.843	1.803	0.28
Initial rhythm asystole	0.336	0.23	0.492	<0.001

Multivariable logistic regression was adjusted for age, sex, bystander CPR, public AED shocks, rescue team defibrillation, adrenaline administration, and initial rhythm. OR = odds ratio; CI = confidence interval; ROSC = return of spontaneous circulation; CPR = cardiopulmonary resuscitation; AED = automated external defibrillator; VF = ventricular fibrillation; VT = ventricular tachycardia; PEA = pulseless electrical activity.

When pupillary diameter was analyzed categorically, patients with very small pupils (1–2 mm) had the highest ROSC rate (42.5 %, OR 1.28, 95 % CI 0.97–1.68), while the reference group (3–4 mm) achieved ROSC in 36.2 %. Progressively larger diameters were associated with sharply reduced ROSC rates: 28.4 % for 5–6 mm (OR 0.69, 95 % CI 0.61–0.79; *p* < 0.001), 12.3 % for 7 mm (OR 0.29, 95 % CI 0.22–0.38; *p* < 0.001), and only 3.6 % for 8 mm (OR 0.09, 95 % CI 0.04–0.19; *p* < 0.001) (Table S1).

The overall discriminatory performance of continuous pupil size for failure of ROSC was modest (AUC = 0.595). ROC analysis confirmed this trade-off between sensitivity and specificity. A cut-off of ≥ 4 mm yielded very high sensitivity (0.935) but poor specificity (0.193, FPR 0.807). At ≥ 6 mm, specificity improved to 0.753 with sensitivity of 0.324 (Youden Index 0.208, the maximum observed). Extremely large cutoffs provided near-perfect specificity but very low sensitivity: at ≥ 7 mm, specificity was 0.953 (FPR 0.047) with sensitivity of only 0.069, while at ≥ 8 mm, specificity increased to 0.990 (FPR 0.010) but sensitivity decreased further to 0.016 (Table S2, Fig. 3).

### Association between pupillary diameter and neurological outcomes

In unadjusted analyses, larger pupillary diameter at EMS contact was associated with a lower likelihood of a favorable neurological outcome (CPC 1–2) at 30 days. Each 1-mm increase in diameter corresponded to a 27 % decrease in the odds of a favorable outcome (OR 0.73, 95 % CI 0.61–0.86, *p* < 0.001). This association persisted after multivariable adjustment for age, sex, initial rhythm, bystander CPR, public AED shocks, and adrenaline administration (Table 4).

**Table 4:** Multivariable logistic regression analysis of factors associated with favorable 30-day neurological outcome (CPC 1–2 vs. CPC 3–5).

**Variable**	**Odds Ratio**	**95 % CI**	**p-value**
Female sex	0.56	0.32–0.98	0.041
Age (per year)	0.98	0.97–1.00	0.010
Bystander CPR (yes)	1.55	0.97–2.46	0.067
Public AED shocks	1.48	0.94–2.34	0.092
Initial ECG (PEA)	0.31	0.06–1.73	0.182
Initial ECG (asystole)	0.26	0.14–0.49	<0.001
Initial ECG (other)	0.06	0.02–0.17	<0.001
Rescue team defibrillation	1.18	1.02–1.37	0.024
IV access (yes)	0.89	0.45–1.78	0.747
Adrenaline (yes)	0.42	0.19–0.92	0.029
Hospital ROSC	12.21	7.27–20.51	<0.001
Pupillary diameter (per mm)	0.73	0.61–0.86	<0.001

Multivariable logistic regression was adjusted for age, sex, bystander CPR, public AED shocks, rescue team defibrillation, adrenaline administration, and initial rhythm. CPC = Cerebral Performance Category; CI = confidence interval; CPR = cardiopulmonary resuscitation; AED = automated external defibrillator; ECG = electrocardiogram; PEA = pulseless electrical activity; IV = intravenous; ROSC = return of spontaneous circulation.

Among patients who achieved prehospital ROSC (*n* = 964), CPC at 30 days was available for 863 patients; in this subgroup, larger pupillary diameter at EMS contact remained independently associated with lower odds of favorable 30-day neurological outcome (CPC 1–2) (Table S3).

### Impact of pupillary light reflex (PLR)

When stratified by pupillary light reflex (PLR), similar trends were observed. Among patients with available PLR data, 3241 (53.3 %) had absent PLR and 2845 (46.7 %) had present PLR. In patients without PLR, the AUC for predicting failure to achieve ROSC was 0.582, with thresholds of ≥7 mm and ≥8 mm achieving specificity of 0.951 and 0.990, but sensitivities of only 0.074 and 0.017, respectively. In contrast, when PLR was present, the AUC improved modestly (0.669). Thresholds of ≥7 mm and ≥8 mm yielded specificities of 0.961 and 1.000, corresponding sensitivities of 0.044 and 0.004 (Table 5). Very large pupils (≥7–8 mm) in the field were associated with failure to achieve ROSC, although the sensitivity was extremely low.

**Table 5:** Diagnostic performance of pupillary diameter cutoffs (4–8 mm) for predicting failure to achieve prehospital ROSC, stratified by pupillary light reflex (PLR) status.

**Analysis group**	**AUC**	**Cutoff**	**Specificity**	**FPR**	**Sensitivity**
Overall (all patients)	0.595	≥7 mm	0.931	0.069	0.187
		≥8 mm	0.992	0.008	0.069
PLR absent	0.582	≥7 mm	0.951	0.049	0.074
		≥8 mm	0.99	0.01	0.017
PLR present	0.669	≥7 mm	0.961	0.039	0.044
		≥8 mm	1	0	0.004

ROSC = return of spontaneous circulation; AUC = area under the ROC curve; ROC = receiver operating characteristic; FPR (false positive rate) = 1 − specificity; PLR = pupillary light reflex.

### Sensitivity and subgroup analyses

Subgroup analyses stratified by PLR status demonstrated that the prognostic effect of pupillary diameter was more pronounced in patients with absent PLR at EMS contact. Extreme value analyses (<2 mm or >6 mm) yielded similar results, suggesting robustness against potential misclassification bias from manual measurement.

## Discussion

This large multicenter study is the first in Japan to demonstrate that greater pupillary diameter at EMS contact is an independent predictor of poor neurological outcome at 30 days in OHCA patients.^6^, ^16^, ^17^ These results support the clinical value of pupillary examination not only in hospital but also in prehospital settings.^7^, ^10^ While neurological prognostication has classically relied on in-hospital findings after return of spontaneous circulation (ROSC), our findings suggest that pupillary assessment performed by EMS at the scene can provide essential prognostic information much earlier in the care continuum.

Prior studies have shown that the absence of pupillary light reflex (PLR) or the presence of fixed, dilated pupils after cardiac arrest is strongly associated with severe brain injury and poor recovery.^6^, ^8^, ^18^ The pathophysiological rationale for this association is that pupillary dilation and loss of reactivity reflect brainstem hypoperfusion and herniation, which occur rapidly in the setting of prolonged global ischemia. Automated infrared pupillometry, which minimizes observer variability and allows quantitative assessment, has been shown to improve the accuracy of neurological prognostication in both post-resuscitation and neurocritical care settings.^7^, ^9^, ^10^ However, most previous work has focused on in-hospital measurements after ROSC, leaving a gap regarding the utility of prehospital assessments.^5^, ^8^

Our findings fill this gap by demonstrating a strong and independent relationship between prehospital pupillary diameter and 30-day neurological outcomes. This association remained robust after controlling for established predictors such as age, initial rhythm, bystander CPR, and use of adrenaline. When restricting the analysis to patients who achieved ROSC, larger pupillary diameter also continued to show an independent association with poor 30-day neurological outcomes (Table S3). This reduces bias from including patients who never achieved ROSC in the denominator and provides stronger evidence that pupillary diameter is a robust prognostic indicator after out-of-hospital cardiac arrest. This suggests that pupillary measurements recorded by EMS can contribute meaningfully to risk stratification even in the chaotic out-of-hospital environment. However, because these measurements are obtained visually, reliability must be interpreted cautiously. In Japan, EMS providers undergo standardized national training and document pupil size on ambulance records using 1-mm increments, sometimes aided by gauge charts with 0.5-mm subdivisions. Despite these safeguards, observer variability remains an inherent limitation, and future validation with automated pupillometry is warranted.^3^, ^19^ For EMS systems in Japan and worldwide, pupillary examination may represent a useful and feasible component of early neurologic assessment, but its role should be confirmed by prospective validation, ideally with automated pupillometry.

Our analysis demonstrates that prehospital pupillary diameter is an independent predictor of ROSC, even after adjusting for key Utstein variables. In multivariable logistic regression, each 1-mm increase in pupil size significantly reduced the odds of ROSC (OR 0.694, 95 % CI 0.644–0.748; *p* < 0.001), a finding consistent with prior observations that fixed or dilated pupils at EMS contact strongly predict poor resuscitation success.^6^, ^16^ The possibility that the observed association between adrenaline administration and ROSC reflects confounding by indication rather than a causal benefit is also noteworthy. Complementary categorical analyses confirmed a stepwise decline in ROSC rates as pupil diameter increased, with patients with very small pupils (1–2 mm) having the most favorable outcomes, and those with larger pupils (≥5 mm) demonstrating progressively lower chances of ROSC (Table S1).

ROC-based threshold analyses highlighted that extremely large pupils (≥7–8 mm) were particularly informative, offering high specificity for predicting resuscitation failure (specificity 0.953 and 0.990, respectively), although sensitivity remained poor (0.069 and 0.016). The clinical implications of pupillary thresholds and PLR stratification suggest that extremely large pupils (≥7–8 mm) may serve as futility markers, although sensitivity remains poor. These findings align with reports that extreme pupillary dilation serves as a strong futility marker, though not sufficient for stand-alone prognostication.^8^, ^18^ Stratification by PLR confirmed this pattern, with absent PLR associated with slightly improved discrimination, consistent with prior work suggesting that combined pupillary size and reactivity may enhance early prognostication.^5^, ^11^ Furthermore, our supplementary analyses suggest that PLR status may modify the effect of pupillary diameter on outcomes. Specifically, the association between larger pupil size and failure to achieve ROSC was more pronounced among patients without PLR, consistent with prior reports that combining pupillary size and reactivity enhances early prognostication.^5^, ^11^ Taken together, these results indicate that pupillary size—especially extreme dilation—can serve as a clinically relevant indicator of futility, but should always be interpreted within a multifactorial framework.

These supplementary analyses indicate that very large pupillary diameters may serve as useful markers of futility but cannot be relied upon as stand-alone criteria. Instead, pupillary size should be interpreted within a broader, multifactorial prehospital assessment framework. By contrast, associations with 30-day neurological outcome should be interpreted with greater caution, as post-ROSC factors such as in-hospital treatment, secondary complications, and withdrawal-of-care practices exert substantial influence on long-term prognosis.^2^

From a practical perspective, incorporating pupillary diameter measurement into EMS protocols offers several advantages. First, it is a non-invasive, rapid, and universally accessible technique requiring minimal training and equipment. Second, it provides real-time feedback about cerebral function when other neurological signs may be difficult to assess. Third, when combined with other prehospital data such as initial ECG rhythm and witnessed status, pupillary assessment may help EMS teams identify patients who would benefit from advanced interventions (e.g., targeted temperature management, extracorporeal CPR) and inform prehospital triage decisions.^2^, ^3^ Furthermore, early communication of pupillary findings to the receiving hospital can enhance multidisciplinary care coordination and allow for earlier family counseling.

Nevertheless, our study highlights several challenges that must be considered before broad implementation. Environmental factors such as lighting, patient positioning, and medications (e.g., atropine, sedatives, neuromuscular blockers) can all influence pupillary measurements.^7^, ^10^ While our analysis relied on manual measurement, future research should evaluate the feasibility and utility of automated infrared pupillometry devices in pre-hospital environments, which have shown promise in hospital-based studies.^5^, ^10^ Additionally, interobserver variability remains a concern, especially in high-stress or low-light conditions. Standardized training for EMS providers, use of decision aids or checklists, and periodic performance feedback may help to mitigate these challenges.

Our data also prompt consideration of the broader implications for prognostication in cardiac arrest care. As resuscitation science evolves, there is increasing recognition that outcome prediction should be multimodal, incorporating clinical examination, physiologic data, and, where possible, biomarkers or imaging.^2^, ^5^, ^20^ Prehospital pupillary diameter represents one important—but not solitary—component of this approach. Future research should focus on integrating prehospital pupillary data with other variables, such as end-tidal CO2, EEG patterns, and early imaging, to develop robust prognostic models that span the entire patient care pathway.

The generalizability of our findings is supported by the large, multicenter SOS-KANTO cohort, which encompasses diverse EMS systems and patient populations across Japan. However, international studies will be required to confirm that these results hold in different health systems, with varying EMS training, protocols, and equipment. Ultimately, the early identification of patients with poor neurological prognosis could help to tailor resource use, minimize futile interventions, and support ethical decision-making regarding ongoing care.

Limitations: Several important variables—including the exact timing of pupil measurement relative to chest compressions, ROSC, or pauses; ambient light conditions; drug administration; and inter-observer variability—were not uniformly documented. Although cases explicitly recorded as post-ROSC assessments were excluded, the registry did not uniformly capture the exact timing of pupil measurement relative to CPR cycles or ROSC. Therefore, despite our exclusion criteria, some residual misclassification regarding measurement timing cannot be entirely ruled out. Pupillary diameter was assessed visually by EMS personnel rather than by automated pupillometry, introducing the possibility of measurement variability. Furthermore, because pupillary diameter was not always recorded bilaterally, potential effects of pupillary asymmetry (e.g., anisocoria) could not be evaluated. In addition, several clinically important covariates, such as the quality and timing of chest compressions, airway management, and in-hospital treatments, were not uniformly captured in the registry and therefore could not be incorporated into the models. Although EMS providers in Japan are trained in neurological assessment and use a nationally standardized ambulance record form specifying diameters in 1-mm increments from 1 to 8 mm, values are inherently estimated rather than directly measured. In some regions, printed pupillary gauge charts with 0.5-mm increments are provided as visual aids, but for registry purposes, all measurements are rounded to the nearest 1 mm. This uniform categorization, intentionally adopted by the SOS-KANTO registry, was designed to reduce false precision and enhance comparability across regions. While pupillary examination is a routine and rapid component of the EMS neurological assessment, its precise timing and potential influence on resuscitation workflow cannot be fully determined from the registry data. Although bilateral measurements were available for most patients, systematic assessment of pupillary asymmetry was not captured as a dedicated variable, precluding formal evaluation of anisocoria and its implications. Finally, our findings are derived from the Japanese EMS system, and caution is warranted when extrapolating to other international contexts. Despite these limitations, the consistency and strength of associations observed—particularly for ROSC and 30-day neurological outcomes—support the robustness of our conclusions. Prospective validation, ideally incorporating automated pupillometry and standardized timing protocols, will be essential to confirm these findings.

## Conclusion

In this large multicenter study, prehospital pupillary diameter was independently associated with both ROSC and 30-day neurological outcomes after out-of-hospital cardiac arrest. These findings highlight the potential value of pupillary assessment as a simple, non-invasive, and universally available tool for early risk stratification in the prehospital setting. However, pupillary size alone has limited discriminatory power and should be interpreted within a multifactorial framework that includes other clinical indicators. Future research using standardized, automated pupillometry is warranted to validate these results, reduce observer variability, and determine how pupillary assessment can best be integrated into prehospital decision-making and prognostic models.

## CRediT authorship contribution statement

**Munekazu Takeda:** Writing – review & editing, Supervision, Methodology, Conceptualization. **Ryokan Ikebe:** Writing – original draft, Visualization, Formal analysis, Data curation. **Takuya Oshiro:** Validation, Resources, Investigation. **Mizuho Namiki:** Writing – review & editing, Supervision, Methodology. **Shimpei Asada:** Writing – review & editing, Validation, Investigation. **Shusuke Mori:** Writing – review & editing, Writing – original draft, Visualization, Supervision, Project administration, Data curation.

## Ethics approval

This study was approved by the institutional review board of Tokyo Women’s Medical University (Approval No. 5242). The study was conducted as a multi-institutional observational study using anonymized data, and informed consent was waived in accordance with the Ethical Guidelines for Medical and Health Research Involving Human Subjects.

## Funding

This research received no external funding.

## Declaration of competing interest

The authors declare that they have no conflicts of interest relevant to this study.
