# Corrigendum to “Association of prehospital pupillary diameter with return of spontaneous circulation and neurological outcome after out-of-hospital cardiac arrest: A multicenter retrospective analysis” [Resusc. Plus 26 (2025) 101112]

**DOI:** 10.1016/j.resplu.2026.101313

**Published:** 2026-04-11

**Authors:** Munekazu Takeda, Nobuya Kitamura, Takashi Tagami, Ryokan Ikebe, Takuya Oshiro, Mizuho Namiki, Shimpei Asada, Shusuke Mori, the SOS-KANTO 2017 Study Group

**Affiliations:** a Department of Emergency and Critical Care Medicine, Tokyo Women’s Medical University, 8-1, Kawada-cho, Shinjuku-ku, Tokyo 162-8666, Japan; b Department of Emergency and Critical Care Medicine, Kimitsu Chuo Hospital, Chiba, Japan; c Department of Emergency and Disaster Medicine, The Jikei University School of Medicine, Tokyo, Japan

The authors regret that the authorship of the published article was incomplete.

The following authors were unintentionally omitted from the original publication: **Nobuya Kitamura** and **Takashi Tagami**.

The corrected author list is updated as above.

This correction has been made in accordance with the authorship policy of the SOS-KANTO 2017 registry.

All authors have agreed to this correction.

**CRediT authorship contribution statement**

**Munekazu Takeda:** Writing – review & editing, Supervision, Methodology, Conceptualization.

**Ryokan Ikebe:** Writing – original draft, Visualization, Formal analysis, Data curation.

**Takuya Oshiro:** Validation, Resources, Investigation.

**Mizuho Namiki:** Writing – review & editing, Supervision, Methodology.

**Shimpei Asada:** Writing – review & editing, Validation, Investigation.

**Shusuke Mori:** Writing – review & editing, Writing – original draft, Visualization, Supervision, Project administration, Data curation.

**Nobuya Kitamura:** Conceptualization, Methodology, Supervision.

**Takashi Tagami:** Conceptualization, Methodology, Supervision.

  All authors approved the final version of the manuscript.

The authors would like to apologise for any inconvenience caused.

