# Supplementary material for: Association of prehospital pupillary diameter with return of spontaneous circulation and neurological outcome after out-of-hospital cardiac arrest: A multicenter retrospective analysis
Source: Resusc Plus. 2025 Sep 26;26:101112. doi: 10.1016/j.resplu.2025.101112 (PMC12547821; doi:10.1016/j.resplu.2025.101112)
Supplement: Supplementary Data 1 [file mmc1.docx]

| **Table S1. Prehospital ROSC rates according to pupillary diameter categories at EMS contact** | | | | | |
| --- | --- | --- | --- | --- | --- |
| **Pupillary Diameter Category (mm)** | **N** | **ROSC Achieved (%)** | **Odds Ratio (vs 3–4 mm)** | **95% CI** | **p-value** |
| **1–2** | 348 | 42.5 | 1.28 | 0.97–1.68 | 0.084 |
| **3–4** | 2143 | 36.2 | 1 | Ref | – |
| **5–6** | 4462 | 28.4 | 0.69 | 0.61–0.79 | <0.001 |
| **7** | 688 | 12.3 | 0.29 | 0.22–0.38 | <0.001 |
| **8** | 110 | 3.6 | 0.09 | 0.04–0.19 | <0.001 |
| ROSC = return of spontaneous circulation; EMS = emergency medical services; CI = confidence interval. | | | | | |

| **Table S2. Diagnostic performance of pupillary diameter cutoffs (4–8 mm) for predicting failure to achieve prehospital ROSC** | | | | | | | | |
| --- | --- | --- | --- | --- | --- | --- | --- | --- |
| **Cutoff (mm)** | **TP** | **FP** | **TN** | **FN** | **Sensitivity** | **Specificity** | **FPR** | **Youden Index** |
| **4** | 6493 | 583 | 139 | 448 | 0.935 | 0.193 | 0.807 | 0.105 |
| **5** | 5420 | 447 | 275 | 1521 | 0.781 | 0.381 | 0.619 | 0.162 |
| **6** | 2248 | 178 | 544 | 4693 | 0.324 | 0.753 | 0.247 | 0.208 |
| **7** | 482 | 34 | 688 | 6459 | 0.069 | 0.953 | 0.047 | 0.118 |
| **8** | 112 | 7 | 715 | 6829 | 0.016 | 0.99 | 0.01 | 0.061 |
| Diagnostic performance of pupillary diameter cutoffs (4–8 mm) for predicting failure to achieve prehospital ROSC (positive condition). TP = true positives (failed ROSC with diameter ≥ cutoff); FP = false positives (achieved ROSC with diameter ≥ cutoff); TN = true negatives (achieved ROSC with diameter < cutoff); FN = false negatives (failed ROSC with diameter < cutoff). Sensitivity = TP/(TP+FN); Specificity = TN/(TN+FP); FPR (false positive rate) = FP/(FP+TN) = 1 − Specificity; Youden index = Sensitivity + Specificity – 1; ROSC = return of spontaneous circulation. | | | | | | | | |

| **Table S3. Multivariable logistic regression analysis of factors associated with favorable 30-day neurological outcome (CPC 1–2 vs. CPC 3–5) among patients who achieved prehospital ROSC** | | | |
| --- | --- | --- | --- |
| **Variable** | Odds Ratio | 95% CI | p-value |
| **Sex (female vs male)** | 0.47 | 0.298–0.742 | 0.001 |
| **Age (per year)** | 0.962 | 0.951–0.973 | <0.001 |
| **Bystander CPR (yes vs no)** | 1.638 | 1.134–2.367 | 0.009 |
| **Public AED use (yes vs no)** | 1.036 | 0.729–1.473 | 0.843 |
| **Number of EMS personnel** | 1.13 | 0.505–2.527 | 0.766 |
| **Pupil diameter (per 1 mm)** | 0.522 | 0.445–0.611 | <0.001 |
| Multivariable logistic regression was adjusted for sex, age, bystander CPR, public AED use, number of EMS personnel, and pupil diameter at EMS contact. Of 9,909 patients in the SOS-KANTO 2017 registry, 964 achieved prehospital ROSC, among whom 30-day CPC data were available for 863 patients. Complete-case analysis was performed, and cases with missing values in covariates were excluded. CPC = Cerebral Performance Category; ROSC = return of spontaneous circulation; CI = confidence interval; CPR = cardiopulmonary resuscitation; AED = automated external defibrillator; EMS = emergency medical services. | | | |
